# Stability studies with tigecycline in bacterial growth medium and impact of stabilizing agents

**DOI:** 10.1007/s10096-020-03970-0

**Published:** 2020-07-27

**Authors:** Lisa F. Amann, Emilia Ruda Vicente, Mareike Rathke, Astrid Broeker, Maria Riedner, Sebastian G. Wicha

**Affiliations:** 1 Department of Clinical Pharmacy, Institute of Pharmacy, University of Hamburg, Bundesstraße 45, DE-20146 Hamburg, Germany; 2 Department of Chemistry, University of Hamburg, Martin-Luther-King-Platz 6, 20146 Hamburg, Germany

**Keywords:** Tigecycline, Stability, Degradation, Mueller Hinton broth, Time-kill studies

## Abstract

**Purpose:**

This study aimed to examine the degradation of tigecycline in Mueller Hinton broth (ca-MHB), as knowledge about bacterial susceptibility is key for therapeutic decisions.

**Methods:**

Antioxidative stabilizers were evaluated on tigecycline stability in a quantitative chromatography assay and tigecycline induced kill against *Staphylococcus aureus* (ATCC29213) was determined in time kill studies.

**Results:**

Ascorbic acid caused rapid degradation of tigecycline and resulted in loss of antibacterial activity. Tigecycline was stabilized in aged broth by 2% pyruvate and bacterial growth, and tigecycline killing was similar to fresh broth without supplementation, but independent of age.

**Conclusion:**

Our results underline the importance of using freshly prepared ca-MHB or the need for stabilizers for tigecycline susceptibility testing while using aged ca-MHB.

**Electronic supplementary material:**

The online version of this article (10.1007/s10096-020-03970-0) contains supplementary material, which is available to authorized users.

## Introduction

Tigecycline is a broad-spectrum antibiotic and indicated for complicated skin and intra-abdominal infections, as well as community-acquired pneumonia [1]. Tigecycline is known to be light and oxygen sensitive [2, 3]. A growth medium age–related effect on tigecycline stability was described, probably mediated by the amount of dissolved oxygen, which can lead to inconsistencies in MIC values [4]. A novel formulation stabilized tigecycline up to 7 days by adding 0.3% ascorbic acid and 6% pyruvate as oxygen-reducing agents [2]. Nevertheless, these additives were only tested in saline and antibacterial activity was determined with *Escherichia coli* in Oxyrase-treated Mueller Hinton broth to remove the dissolved oxygen. Hence, the degradation kinetics of tigecycline and potential impact of bacterial killing in aged vs. fresh cation-adjusted Mueller Hinton broth (ca-MHB) and the impact of stabilizing agents remain unknown.

The objective of this study was (i) to quantify the degradation process in fresh and aged ca-MHB using different stabilizing agents and (ii) to evaluate their impact on bacterial growth and tigecycline-induced kill, to derive recommendations for consistent in vitro susceptibility testing of tigecycline.

## Material and methods

### Materials

Lyophilized powder of tigecycline was obtained from Pfizer (New York, United States of America, LOT: J49085). *Staphylococcus aureus* (ATCC29213) was obtained from the American Type Culture Collection (Manassas, Virginia, USA). All other chemicals were purchased from Sigma-Aldrich.

### Stability assay

#### Preparation of tigecycline samples

A stock solution of tigecycline (1.0 mg/mL) was prepared in 0.9% saline solution and diluted to 10 μg/mL with fresh (< 12 h) or aged (up to 7 days at 4 °C or room temperature) ca-MHB, with and without stabilizing agents, incubated at 37 °C for 24 h, protected from light stored in a Eppendorf vial rack. Stock solutions of stabilizing agents were adjusted to pH 7.0 and spiked to ca-MHB after storing. Aged ca-MHB, stored at 4 °C, spiked with 2% pyruvate (ca-MHB_7days_p2%_), as well as the combination of 6% pyruvate and 0.3% ascorbic acid were tested (ca-MHB_7days_p6% + aa0.3%_). Ca-MHB spiked with 0.3% ascorbic acid was tested in freshly prepared ca-MHB (ca-MHB_aa0.3%_). Furthermore, we investigated tigecycline stability in non-supplemented ca-MHB stored 7 days at room temperature (ca-MHB_7days_RT_) versus supplementation with 2% or 6% pyruvate. The pH of ca-MHB was measured and equivalent to the manufacturer given value (pH 7.3).

#### HPLC analysis

Calibration curve, quality control, and samples were measured by UHPLC (Ultimate 3000 SD Dionex, Softron GmbH, Germering, Germany) equipped with a Nucleoshell RP 18 (MachereyNagel, Dueren, Germany) using UV detection at 350 nm. Samples containing 0.3% ascorbic acid reached the lower limit of quantification. Therefore, a QTRAP 5500 mass spectrometer (SCIEX, Framingham, Massachusetts, USA) coupled with a 1290 Infinity HPLC II (Agilent Technologies, California, USA) was used to quantify tigecycline in these samples. A detailed description of the analytical method is described in Supplement Text 1.

#### Preparation of standards and quality control

For each measurement, a calibration from 0.1 to 10 mg/L using seven calibrators and double determination was prepared. Two independently prepared quality controls were analyzed in each run with a high and a low concentration of tigecycline. The inaccuracy and imprecision of the assay across all analytical runs was < 12% and < 4%, respectively.

### Time-kill studies

The effect of stabilizing agents on bacterial growth was tested with *Staphylococcus aureus* (ATCC29213), as recommended by CLSI [5] in three settings: (i) fresh and aged ca-MHB without adjuvants, (ii) ca-MHB_aa0.3%_, or (iii) ca-MHB_7days_p2%_. Stock solutions of ascorbic acid (25%) were adjusted to pH 7 and spiked to aged ca-MHB to obtain a final concentration of 0.3%. The pH of the spiked ca-MHB was not altered in the presence of the stabilizing agents and identical to the value given by the manufacturer (pH 7.3). The “reference” MIC was tested in freshly prepared broth according to the CLSI guideline before the experiment [5].

Time kill curves were determined in *n* = 2 at an initial inoculum of 10^6^ CFU/mL and incubated for 120 min at 37 °C to logarithmic growth phase before the antibiotic was added. Tigecycline concentrations of 0.5× MIC (0.063 μg/mL), 1× MIC (0.125 μg/mL), 2× MIC (0.25 μg/mL), 4× MIC (0.5 μg/mL), and 8× MIC (1 μg/mL), as well as growth controls were studied over 24 h. The resulting MIC was determined visually, at 24 h, in presence of stabilizing agents or in aged ca-MHB, evaluating turbidity of testing solution alongside the time kill studies.

## Results

### Stability studies

The stability of tigecycline in fresh and aged ca-MHB with and without adding stabilizing agents was investigated (Table 1). In 7-day-old, non-stabilized ca-MHB, a recovery of 80.1% was found within 24 h. Conversely, in fresh, non-stabilized ca-MHB, 99.6% tigecycline was measured. Moreover, tigecycline degraded already to a remaining concentration of 89.7% using the same ca-MHB solution 1 day after preparation. The stabilizing agents had various effects: In ca-MHB_aa0.3%_, tigecycline degraded rapidly and only 4.7% remained in a freshly prepared solution within 24 h. In ca-MHB_7days_p2%_, we observed a stabilizing effect and 97.1% were recovered. Moreover, the combination of 6% pyruvate and 0.3% ascorbic acid, as recommended by *Jitkova* et al. [2], was inferior to 2% pyruvate alone, and a remaining concentration of 97.2%, if freshly prepared, and 95.1% in ca-MHB_7days_p6% + aa0.3%_ was measured. Furthermore, the stability of tigecycline was investigated in ca-MHB_7days_RT_ and the age dependency of tigecycline stability was even more observable. In MHB_7days_RT_ with  2% pyruvate, a remaining concentration of 87.8% was measured (Table 1), whereas in non-stabilized ca-MHB_7days_RT_, only 27.9% tigecycline were recovered. As described above, the combination of 0.3% ascorbic acid and 6% pyruvate was not superior to pyruvate alone so that pyruvate was increased to 6% to enhance tigecycline stability in MHB_7days_RT_ and 97.1% could be recovered after 24 h.

**Table 1:** Tigecycline concentrations expressed as a relative percentage of the initially measured tigecycline concentrations (= 100%) in cation-adjusted Mueller Hinton broth (ca-MHB) with or without supplementation, incubated in the dark at 37 °C over 24 h stored in Eppendorf vial racks. Ca-MHB age of 0 days was defined as ca-MHB preparation less than 12 h before the experiment started. Ca-MHB was stored up to 7 days at 4 °C or at room temperature. Storage conditions and age refer to Ca-MHB without supplements in absence of tigecycline before the 24 h incubation period with tigecycline with and without supplements at 37 °C was initiated

Matrix	Age (days)	Storage cond.	Recovery at 24 h(%)	Range (%)	*p* value
Ca-MHB	0		99.6	94.5–103.5	0.412
	1	4 °C	89.7	87.4–94.8	0.400 · 10^−2^
	7	4 °C	80.1	82.8–75.6	2.44 · 10^−6^
	7	Room temperature	27.9	25.9–30.0	2.2 · 10^−16^
Ca-MHB + 0.3% ascorbic acid	0		4.7	3.5–5.1	2.47 · 10^−6^
Ca-MHB + 2% pyruvate	0		98.8	97.0–100.3	9.20 · 10^−2^
	7	4 °C	97.1	96.4–99.6	8.40 · 10^−2^
	7	Room temperature	87.8	86.6–88.8	1.69 · 10^−11^
Ca-MHB + 0.3% ascorbic acid+6% pyruvate	0		97.2	93.4–100.2	0.079
	7	4 °C	95.1	91.3–97.3	6.40 · 10^−2^
Ca-MHB + 6% pyruvate	7	Room temperature	97.1	95.6–98.5	1.1 · 10^−2^

### Time kill studies

**Fig. 1:**
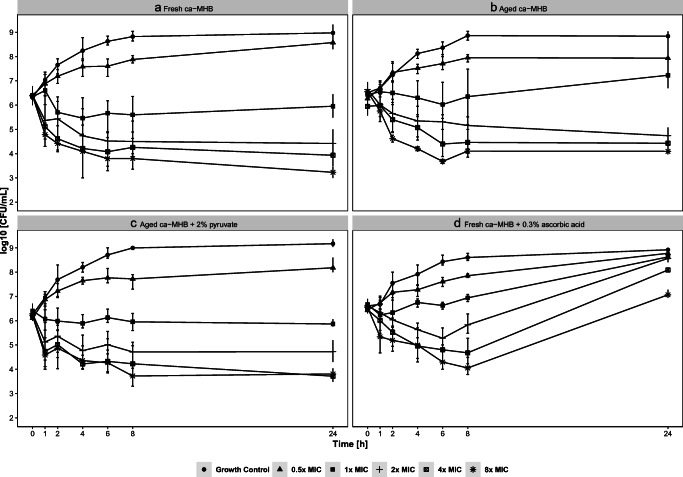
Time kill curves: colony-forming units (CFU) per mL over time by MIC (minimal inhibitory concentration) of tigecycline. The reference MIC was determined according the CLSI guideline using freshly prepared ca-MHB and was 0.125 mg/L. Error bars denote range of minimum to maximum value. All experiments were carried out in duplicates. **A** Freshly prepared ca-MHB. **B** Aged ca-MHB (7 days at 4 °C). **C** Aged ca-MHB (7 days at 4 ° C) supplemented with 2% pyruvate. **D** Fresh ca- MHB containing 0.3% of ascorbic acid

Time kill curves were conducted to investigate the impact of tigecycline degradation on observed pharmacodynamic effects (Fig. 1). The results show that neither ascorbic acid (*p* = 0.410) nor pyruvate (*p* = 0.161) affected the natural growth in absence of tigecycline, compared to non-supplemented ca-MHB. Using fresh ca-MHB, > 1.5-log killing at > 2× MIC and moderate killing at 1× MIC were observed after 24 h. In contrast to that, at 1× MIC in 1-week-aged ca-MHB, no killing but a growth to > 1-log higher CFU/mL was observed compared to fresh broth. The growth/killing pattern in ca-MHB_7days_p2%_ was not different from freshly prepared ca-MHB.

The strong degradation process related to 0.3% ascorbic acid translated to substantially reduced killing: Antibacterial activity at 1× MIC ceased 1 h after addition of tigecycline, and regrowth was observed even at 8× MIC.

In fresh or stabilized ca-MHB_7days_p2%_, an MIC of 0.125 mg/L was determined. The use of aged broth led to determination of a MIC of 0.25 mg/L, in ca-MHB_aa0.3%_ > 1 mg/L, respectively, concluded by visible turbidity.

## Discussion

The present study elucidates the stability of tigecycline in the most important bacterial growth medium ca-MHB thereby using state-of-the-art bioanalytical assays as a prerequisite to obtain quantitative stability data in combination with pharmacodynamic studies [6]. Moreover, pyruvate and ascorbic acid as potential stabilizing agents for tigecycline in ca-MHB were comprehensively assessed.

The results of the present study in freshly prepared vs. aged ca-MHB are in line with a previous study [4]. However, our study adds quantitative information as previously, solely the pharmacodynamic age-dependence of ca-MHB was studied [4]. The use of freshly prepared broth might not be possible in routine labs, or is also impractical in long-term in vitro studies such as hollow-fiber experiments. The addition of Oxyrase has been proposed to stabilize tigecycline in broth to reduce the amount of dissolved oxygen to stabilize tigecycline [3]. Yet, Oxyrase represents a costly agent and the herein proposed antioxidative pyruvate is much more economic. Pyruvate might prevent tigecycline’s oxidation at its phenolic group [7] in order to stabilize ca-MHB regardless of the ca-MHB age.

Another important aspect is that stability data generated in saline does not allow inferring about ca-MHB: *Jitkova* et al [2] found that 6% pyruvate in saline resulted in an insufficient stabilization, with a remaining tigecycline concentration of approx. 70% after 24 h. In contrast to that, we found a recovery of 97.1% in ca-MHB_7days_p2%_ after 24 h. The differences for ascorbic acid are even more striking: By solely adding ascorbic acid, we quantified rapid degradation, even though pH control was applied, suggesting that ascorbic acid induces a destabilizing reaction in ca-MHB. A mass spectroscopic full scan could not detect any known degradation product. *Jitkova* et al. found that ascorbic acid alone was also insufficient to fully stabilize tigecycline in saline but did not observe a destabilization as quantified by us. In saline solution, 67.6% of freshly prepared tigecycline were recovered after 3 days [2]. Furthermore, a strong degradation of tigecycline occurs in non-stabilized ca-MHB_7days_RT_ and 2% pyruvate is not sufficient to prevent tigecycline from degradation; hence, 6% pyruvate is needed for stabilization. The broth aging process occurs faster at room temperature, so that 2% pyruvate cannot conserve tigecycline. Even though ca-MHB_7days_RT_ with 6% pyruvate shows comparable results as ca-MHB_7days_p2%_ stored at 4 °C, we recommend storing the broth at 4 °C and supplement with 2% pyruvate before use, or the use of fresh ca-MHB, to save costs.

The measured kinetic data were consistent the pharmacodynamic effects in our study, i.e., faster and more intense regrowth was observed when tigecycline degraded faster. If ca-MHB age is not controlled, a twofold higher MIC value might be found due to tigecycline degradation, which can be avoided by addition of 2% pyruvate. The use of ascorbic acid, although stabilizing tigecycline in saline, cannot be recommended in ca-MHB.

## Electronic supplementary material

ESM 1 (DOCX 21 kb).

## Data Availability

Not applicable.
