# Supplementary material for: Stability studies with tigecycline in bacterial growth medium and impact of stabilizing agents
Source: Eur J Clin Microbiol Infect Dis. 2020 Jul 27;40(1):215–8. doi: 10.1007/s10096-020-03970-0 (PMC7782409; doi:10.1007/s10096-020-03970-0)
Supplement: Supplementary file 1 — (DOCX 21 kb). [file 10096_2020_3970_MOESM1_ESM.docx]

Supplementary material to:

Stability studies with tigecycline in bacterial growth medium and impact of stabilizing agents.

Lisa F. Amann^1^, Emilia Ruda Vicente^1^, Mareike Rathke^1^, Astrid Broeker^1^, Maria Riedner^2^, Sebastian G. Wicha^1^

^1^Dept. of Clinical Pharmacy, Institute of Pharmacy, University of Hamburg, Bundesstraße 45, 20146 Hamburg, Germany

^2^Dept. of Chemistry, University of Hamburg, Martin-Luther-King-Platz 6, 20146 Hamburg, Germany

**Supplement Text 1: LC-UV and LC-MS/MS methods**

All samples (200 µL) were processed for protein precipitation by adding 200 µL of ice-cold acidic methanol to reduce adsorption to plastic labware, derived from Dorn et al [1]. The samples were centrifuged afterwards and 10 µL of the supernatant were injected.

Calibration curve, quality control and samples were measured by UHPLC (Ultimate 3000 SD Dionex, Softron GmbH, Germering) equipped with a Nucleoshell RP 18 (100x3 mm, 2.7 µm particle size, MachereyNagel, Dueren, Germany) using UV detection at 350 nm. As mobile phases (A) Milli-Q® water containing formic acid (95:5, v/v) and (B) methanol, acetonitrile, formic acid (47.5:47.5:5, v/v) were used in a gradient program. The gradient conditions were as follows: linear gradient starting at 1% B to 15% B within 3 min, then linear gradient to 70% B over 2 min and then isocratic at 70% B for 3 min. Afterwards, the pumps were programmed to 1% B over 1 min. A 4 min reconditioning time was used before injection of the next sample. Total run time was 13 min at a flow rate of 0.8 mL/min. The retention time of tigecycline was 3.4 min.

A QTRAP 5500 (SCIEX, Framingham, Massachusetts, USA) electrospray ionisation mass spectrometer coupled with an 1290 Infinity HPLC II (Agilent Technologies, California, USA) was used for LC-MS/MS quantification of broth samples containing ascorbic acid, due to reaching the limit of quantification.

For LC-MS/MS solvents were used in LC-MS grade. Separation was performed on a Nucleodur C18 Gravity-SB (100x3 mm, 3µm particle size, MachereyNagel, Dueren, Germany). Solvents (A) water containing formic acid (99.9:0.1, v/v) and (B) methanol, acetonitrile, formic acid (49.9:49.9:0.1, v/v) were used in a gradient program. The gradient conditions were as follows: starting conditions 1% B, linear gradient starting at 1% B to 20% B within 4 min, then linear gradient to 70% B over 2 min and then isocratic at 70% B for 3 min. Afterwards, the pumps were programmed to 1% B over 1 min. A 4 min reconditioning time was used before injection of the next sample. Total run time was 15 min at a flow rate of 0.3 mL/min. The retention time of tigecycline was 6.3 min.

LC–MS/MS data were acquired and analysed using Analyst 1.7 software (SCIEX, Framingham, Massachusetts, USA). The multiple reaction monitoring (MRM) transitions used were m/z 586.3/513.2 (quantifier) and 586.3/569.2 (qualifier).

**References**

1. Dorn C, Kratzer A, Liebchen U, Schleibinger M, Murschhauser A, Schlossmann J, et al. Impact of Experimental Variables on the Protein Binding of Tigecycline in Human Plasma as Determined by Ultrafiltration. J. Pharm. Sci. [Internet] 2018;107:739–44. doi: https://doi.org/10.1016/j.xphs.2017.09.006doi: 10.1016/j.xphs.2017.09.006
